# A long way to go: engagement of men and boys in country family planning commitments and implementation plans

**DOI:** 10.12688/gatesopenres.13230.1

**Published:** 2021-05-21

**Authors:** Christopher Hook, Karen Hardee, Tim Shand, Sandra Jordan, Margaret E. Greene

**Affiliations:** 1 Promundo-US, Washington, DC, 20036, USA; 2 What Works Association, Washington, DC, 20036, USA; 3 ShandClarke Consulting Ltd, London, UK; 4 Public Health Consultant, Washington, DC, 20036, USA

**Keywords:** family planning, contraception, male engagement, gender equality, FP2020, FP2030, sexual and reproductive health and rights

## Abstract

**Background:** Evidence shows that, to reach global goals related to women and girls’ access to modern family planning (FP) and gender equality, it is critical to understand and account for the role of men and boys as users of reproductive health services, as partners for millions of women & girls around the world, and as advocates in their communities. Under the Family Planning 2020 (FP2020) partnership, countries were encouraged to develop costed implementation plans and action plans in an effort to provide 120 million additional women and girls with contraception. As FP2020 becomes FP2030, reviewing these previously-developed strategies helps understand the extent to which countries considered the engagement of men as an important aspect of their family planning portfolios.

**Methods: **We conducted textual analysis on commitments and implementation plans related to achieving FP2020 commitments in seven countries in Africa and one in Asia to determine the extent to which male engagement was incorporated into country or subnational family planning goals, with particular focus on FP policy, program, and financial commitments.

**Results: **Some of the documents analyzed included robust plans for including male engagement in their efforts to expand access to FP.  The strongest aspects of male engagement programming were those that sought to engage men as advocates for women’s access to and use of FP services, and improve men’s knowledge and attitudes related to contraception and reproduction. The weakest aspects were engaging men as users of services and, vitally, tackling underlying gender norms which hamper men’s and women’s health-seeking behaviors and attitudes.

**Conclusions: **Developing FP programs that target men and boys as people deserving of reproductive health services, as partners with women in building their families, and as social activists in their communities, will complement and strengthen existing FP programs as well as promote broader goals related to gender equality.

## Introduction

In 2012, the organizers of the London Summit for Family Planning – the Bill & Melinda Gates Foundation, the UK Department for International Development ^i^ (DFID), and core conveners the United States Agency for International Development (USAID) and the United Nations Population Fund (UNFPA) set an ambitious goal of expanding access and enabling use of contraceptive use by an additional 120 million women and girls in the world’s poorest countries by 2020 ( Brown *et al*., 2014). In 2015 the 2030 Agenda for Sustainable Development (SDG) included a target of universal access to reproductive health. Following on from Family Planning 2020 (FP2020), the partnership to implement the 2012 goal, the Family Planning 2030 (FP2030) partnership has set its vision as:

“Working together for the future where all women and adolescent girls everywhere have the freedom and ability to make their own informed decisions about using modern contraception and whether or when to have children, lead healthy lives, and participate as equals in society and its development.”

These goals have largely maintained the field’s traditional focus on women and girls, yet attention to gender at the 1994 International Conference on Population and Development (ICPD) in Cairo resulted in a renewed call to involve men more actively in reproductive health ( Boender *et al*., 2004; Ringheim, 1999), primarily as partners to support the autonomous decisions of women ( Wentzell & Inhorn, 2014). FP2030 notes as one of its Guiding Principles that “Positive male inclusion is needed to truly transform normalization of family planning and at the same time share the burden of the decisions and implications of family planning.” Over the years, the vision for constructive male engagement in family planning and reproductive health has evolved from encouraging men to be supportive partners of women’s reproductive health decisions, to also encouraging men to be change agents in families and communities, as well as meeting their own reproductive health needs ( IGWG, 2009). Evidence shows that engaging men improves reproductive health and gender equality outcomes ( Adelekan *et al*., 2014; Ashfaq & Sidiq, 2015; Boender *et al*., 2004; Greene *et al*., 2011; IRH, 2013; Kraft *et al*., 2014; Rottach *et al.*, 2009; Sikder *et al.*, 2020). Evidence also shows that men have not been particularly well served by family planning programs ( Hardee *et al.*, 2017).

Following the 2012 London Summit on Family Planning, countries were encouraged to make concrete policy, program and financial commitments to improve family planning (FP) uptake. To implement these commitments, countries were called on to develop plans with the intentional mission of empowering women and girls by investing in rights-based family planning. Countries developed costed implementation plans (CIPs), a multi-year actionable roadmap to help governments achieve their FP goals and annual action plans to track their progress in achieving their commitments. In 2017, these governments had the opportunity to renew their commitments, and several new countries joined the FP2020 partnership.

By 2019, 47 countries had created CIPs and associated action plans in efforts to reach their FP goals. The country plans provide a clear description of the ways in which countries anticipate reaching their FP2020 goals. Given the evidence showing the importance of engaging men and boys in FP programming as a means both of achieving increased family planning uptake and transforming unhealthy gender norms ( Doyle *et al*., 2018; Shattuck *et al*., 2011), it is important to ask how donors, implementing partners, and countries are or plan to include this critical approach in future national and sub-national family planning policies and plans, including costed implementation plans to accelerate their FP program planning.

## Methods

Between June 2018 and March 2019, the authors analyzed commitment and implementation plan documents related to FP2020 in six countries in Africa (Democratic Republic of the Congo [DRC], Ethiopia, Kenya, Nigeria [nationally and in the Gombe, Kaduna, and Lagos states], Niger, and Senegal), and one country in Asia (Pakistan’s national commitment and the provincial plan in Sindh). These strategies were developed between 2012 and 2017 and share a similar structure across the selected countries. Among these, 10 are included in this paper. For two countries, namely Kenya and Senegal, two CIPs are included in the analysis. To retain a focus on FP2020 related outputs, other national strategies and plans, such as national reproductive health strategies, were excluded from our analysis. All documents included in this paper are available on the FP2020 website ( www.familyplanning2020.org). The documents included:

- Country and/or sub-national Costed Implementation Plans (CIPs), the primary focus of this analysis
- Country Commitments made at the 2012 London Summit on Family Planning and the 2017 Family Planning Summit
- Country Action Plans designed to provide insight into country’s achievement of their commitments
- Self-Reporting Questionnaires submitted to FP2020 on progress made towards achieving goals

Table 1 shows which documents were available for the countries/sub-national levels included in this paper.

**Table 1.** Documents included in analysis of male engagement in relation to Family Planning 2020 (FP2020), by country.

	Document for Analysis			
Country/sub-national level	Family planning costed implementation plan	Country commitments	Country action plan	Self-reporting questionnaire for FP2020
**Democratic Republic of** **the Congo (DRC)**	Family Planning: National Multisectoral Strategic Plan 2014–2020 (NMSPP) (English)	FP2020 Commitment 2017 (French)	National Action Plan 2016 (French) Workplan 2018–2019 (French)	Questionnaire 2018 (French)
**Ethiopia**	Costed Implementation Plan for Family Planning in Ethiopia, 2015/6–2020	FP2020 Commitment 2012	Country Action Plans 2017–2018, 2018–2019	Annual Self-Reporting Questionnaire - 2018
**Kenya**	National Family Planning Costed Implementation Plan 2012–2016 National Family Planning Costed Implementation Plan 2017–2020	FP2020 Commitment 2012	Country Action Plans 2016– 2017; and 2018–2019	
**Niger**	Planification Familiale au Niger: Plan d’Action 2012–2020 [Family Planning in Niger: Action Plan 2012–2020]	FP2020 Commitment (2012)	National Action Plans 2016–2018, 2018–2019	Annual Self-Reporting Questionnaires (2016, 2018)
**Nigeria – national**	Nigeria FP Blueprint (Scale-Up Plan) 2014–2020	Nigeria FP2020 Commitment (2012)	Nigeria 2018–2019 Actions for Acceleration	Annual Self-Reporting Questionnaire 2018
** Gombe State**	Gombe State Framework for the Implementation of Expanded Access to Family Planning Services 2013–2018			
** Kaduna State**	Costed Implementation Plan for Child Spacing, 2016–2018			
** Lagos State**	Lagos State Family Planning Costed Implementation Plan, 2016–2018			
**Pakistan**	Pakistan did not develop a national CIP. At the time of analysis for this paper only Sindh Province in Pakistan had published its CIP.	Pakistan FP2020 Commitment (2012)		
** Sindh province**	Costed Implementation Plan on Family Planning for Sindh, 2015–2020			
**Senegal**	National Family Planning Action Plan 2012–2015 *Cadre Strategique National de Planification Familiale 2016–2020* *[*National Strategic Framework for Family Planning 2016-2020]	Senegal FP2020 Commitments, 2012 and 2017 (French)	National Action Plans 2016, 2018–2019 (French)	Annual Questionnaires: 2016, 2018 (French)

### Definition of male engagement

Our definition of male engagement draws on a categorization that was developed by Greene *et al*. (2006) and adopted by the Interagency Gender Working Group; our definition emphasizes the need to address gender norms:

The intentional inclusion and participation of men and boys in family planning programs as supportive partners, contraceptive users, and agents of change, with an emphasis on addressing gender norms and power differentials throughout the life-cycle.

### Analytic Framework

Using our definition of male engagement as a guide, and drawing on the FP High Impact Practice Initiative strategic planning guide for engaging men and boys in family planning, a framework on best practices for male engagement in family planning programming was developed in order to analyze these documents. We applied this framework to each of the existing standardized domains within the CIPs, assessing the extent to which they incorporated male engagement, and added new dimensions assessing the extent to which they addressed gender norms and promoted use of male methods. This framework included the following areas:

1) Policy and advocacy: proposed policy and/or standards changes regarding male engagement at community, regional, and/or national levels, as well as providing increased advocacy to decision-makers
2) Financing and governance: proposed increases of resources for male engagement at community, regional or national levels, or changes in governance
3) Demand generation: proposed activities, projects or programs seeking to increase demand for family planning and/or change the health-seeking behavior of FP clients
4) Service delivery and human resources: proposed activities, projects or programs seeking to directly improve the provision of services to men as well as women
5) Research, monitoring & evaluation: proposed research or monitoring and evaluation (M&E) indicators seeking to measure program quality (e.g. percent of men who support the use of modern contraception either for them or their partners).
6) Gender norms: activities, projects or programs seeking to challenge inequitable gender norms across one or more levels of the socioecological model (individual, family, community, societal).
7) Male FP methods: activities, projects or programs seeking to increase use of modern male methods (vasectomy, condoms) and calendar-based methods (e.g. Standard Days Method), undertaking specific strategies to broaden the method mix and increase uptake of male methods *.*
Keyword searches, conducted in English and French, were used to identify relevant content, based on the analytic framework. These keywords are available in the *Extended data* ( Hook, 2021).

## Country analysis

### DRC

The DRC made a commitment to FP2020 in 2017, thus its *National Multisectoral Strategic Plan for 2014–2020* became its CIP. The plan notes that “men are often considered as an obstacle or a barrier to family planning. In many cases, men believe that authorizing women to use family planning will encourage prostitution”. Yet, the plan also cites the 2007 Demographic and Health Survey statistic that seven in 10 men have never received any information regarding family planning. The plan notes that implementation will “ensure a sustained increase in modern contraceptive use by Congolese women and men who wish to use contraception.” Approaches involving men include increasing access for men and women to services in the public and private sectors, constructing youth-friendly service delivery points in each of country’s 516 health zones, training teachers on comprehensive sexuality education, addressing demand creation by developing programs targeting men, especially young men, and widening access to mobile fertility applications that would empower couples to plan their pregnancies. While these approaches are promising, little detail is provided on how they would be implemented. 

### Ethiopia

The *Costed Implementation Plan for Family Planning in Ethiopia, 2015/6–2020* reveals a strong intention to generate demand for sexual and reproductive health (SRH) by improving knowledge, building skills, and decreasing stigma among adolescents, women and men ( Ministry of Health [Ethiopia], 2016). Ethiopia’s CIP notes that SRH is not just a women’s issue:

“Although men share responsibility for reproductive health decisions, lack of a specific focus on them can lead to the belief that family planning is not men’s concern. However, male involvement is crucial to a successful demand creation campaign” (p. 15).

Furthermore, Ethiopia’s CIP states that “specific demand creation efforts will be targeted at youth (ages 15–19), community and religious leaders, and men as husbands and fathers,” including through special regional Men’s Day events to raise men’s awareness of family planning and using male role models to guide group education and conduct community outreach and mobilization. 

Ethiopia’s service delivery improvements focus on decreasing bias and improving provider knowledge and patient interaction, as well as shifting services to become more welcoming to adolescents and improve confidentiality. Ethiopia’s strategies for improving family planning use lack detail about the content of social and behavior change communications to promote FP use, and about how programs engaging men and boys are transformative, i.e. addressing reproductive autonomy and power within relationships. Although the CIP says that FP commodities should be available to both men and women (including youth), male methods (e.g. sterilization and condoms) and male-cooperative methods such as Cyclebeads are not a priority for Ethiopia. Ethiopia’s CIP does not emphasize couples counselling in family planning, and service statistics capture only women’s counseling and service uptake. One process indicator mentions men: “availability of accessible, relevant, and accurate information about SRH, tailored to young men” (p. 36). While Ethiopia’s CIP does note that barriers to contraceptive uptake “include power and gender dynamics that inhibit women from making open decisions about FP use in their households” (p. 15), the CIP is not clear on how these should be addressed. 

### Kenya

Kenya completed its first CIP (2012–2016) and its second CIP (2017–2020). Kenya’s *National Family Planning Costed Implementation Plan 2012–2016* notes that low male involvement has been a traditional barrier to family planning in Kenya and noted the plan to conduct an assessment of male involvement in order to develop a strategy ( Ministry of Health [Kenya], 2012). If this assessment was conducted, it was not made available online. In almost identical language, Kenya’s *National Family Planning Costed Implementation Plan 2017–2020* describes low male involvement in FP and FH services as a cultural barrier ( Ministry of Health [Kenya], 2017). Yet no references to male involvement activities were found in Kenya’s 2017–2020 CIP. Demand generation within underserved groups is central to Kenya’s family planning objectives with particular focus on youth, unmarried sexually active youth, people living in rural areas, and people living with disabilities or HIV/AIDS. The country proposes to reach these groups with in-person mobilization and mass media messaging. However, it is not clear how boys are being targeted differently from girls. Additionally, men are not mentioned in relation to service delivery innovations or any other domains.

### Niger

Niger’s CIP, *Planification Familiale au Niger: Plan d’Action 2012–2020* (Family Planning in Niger: Action Plan 2012–2020) and other family planning documents related to FP2020 focus heavily on engaging gatekeepers who may be opposed to family planning, including members of the government as well as Islamic and traditional leaders ( Republique du Niger, 2012). They also show a commitment to using peer educators and engaging husbands to help address the normative barriers to family planning use at a community level. Other efforts, designed to address myths and misconceptions about family planning in public fora, suggest an effort to reach male decision-makers with convincing messages via television and radio. Importantly, all of these programs have a wide focus, aiming to cover the entire country. 

While the CIP proposes advocacy to and mobilization of gatekeepers and change agents who are presumably male, there is rarely an explicit mention of engaging men; the words “gender” and “male” are not found in the CIP and “men” is found only three times. There is a lack of detail related to demand creation, including, for example: How are gender and power being incorporated into messaging? Who is delivering the messages, and how are they working in conjunction with women? How are norms being addressed with adolescent boys? Demand generation programs targeting young people and men do not include a complementary effort to conduct training and refreshers on such things as youth-friendly and gender sensitized services. Furthermore, no indicators measure male engagement activities.

### Nigeria – national

Nigeria’s CIP, *Nigeria Family Planning Blueprint (Scale-Up Plan) 2014–2020*, calls for the selection of advocates who will engage at the community, state, and national levels, with particular focus across key sectors in government and in the private sector, to serve as family planning champions and to address the norms that undermine family planning access. Nigeria’s CIP also outlines several demand generation activities, including a national communications campaign and the integration of a comprehensive sexuality curriculum into secondary schools with complementary interventions with peer educators. This demand generation will be met through the bolstering of services to young people through the training of providers as well as the creation of youth-friendly spaces in primary health centers. Lastly, Nigeria will track behavioral and attitudinal changes through several key indicators. Male engagement is nevertheless mentioned only once across all of these activities. Nigeria indicates efforts to install youth-friendly service delivery points in primary health centers, but there is no indication of whether the services address the specific needs of both girls and boys. 

### Nigeria - Gombe State

Very little information that focuses on men and boys is provided in the *Gombe State Framework for the Implementation of Expanded Access to Family Planning Services 2013–2018* ( Gombe SMoH, 2012). However, the presence of a brothers for health initiative shows understanding of the importance of male engagement to some extent, though how this will be carried out gets little attention. Youth family planning is clearly a priority, in policy, demand generation, and service delivery spheres. However, there is no explicit description of how youth activities are targeting boys and girls separately. Lastly, while the indicators proposed are limited, two of them track attitudinal change among men and community leaders.

### Nigeria - Kaduna State

In Kaduna State’s *Costed Implementation Plan for Child Spacing, 2016–2018*, policy and advocacy activities related to male engagement include activities to institute youth-friendly services at all Kaduna State clinical facilities, and to introduce reproductive rights awareness in various service providers’ training curricula ( Kaduna State Government, 2016). Demand generation activities in Kaduna State include outreach to men and customary and religious leaders through traditional and social media, as well as through the availability of posters and fact sheets in places like schools and clinics which focus on dispelling myths and reinforcing the importance of family planning for families and the health of the mother and child. To meet the new demand, Kaduna State proposes several new male engagement activities in the service delivery domain, including the aforementioned training, the creation of job aids for gender-sensitive family planning counseling, purchasing supplies and equipment which is important for the bolstering of all 380 facilities as youth-friendly sites, and enabling midwives to train community extension workers on youth-friendly service provision. However, there is little clarity about the content of the demand generation messaging.

### 

### Nigeria - Lagos State

The *Lagos State Family Planning Costed Implementation Plan, 2016–2018* includes limited male engagement activities ( Lagos State Ministry of Health, 2016). There is no evidence of attention to male engagement at policy, financing or governance levels. The stronger focus on engaging men is included within the demand generation activities, which include a new behavior change communication strategy, outreach to cultural and religious leaders, use of male champions, and targeting male adolescents with information. However, the CIP is not specific about what the messaging will be, and how it will challenge inequitable gender norms. Few service delivery improvements are proposed other than strengthening youth-friendly services, although there is no detail about how the services will be gender-responsive. Indicators in Lagos State’s CIP fall short of measuring attitudinal or behavior change among men or couples.

### Pakistan – national

As a result of a 2010 national law, the health and population welfare functions in Pakistan underwent devolution and were fully transferred to the provincial level. While Pakistan made a national pledge at the London Summit on Family Planning in 2012, provinces were tasked with developing their own strategies. Of these, four developed CIPs.

### Pakistan - Sindh province

The *Costed Implementation Plan on Family Planning for Sindh* (2015–2020) emphasizes the pairing of bolstered demand and supply-side activities to attain its FP goals. The need to engage men and youth emerged from the process of prioritization that engaged stakeholders in developing the CIP ( PWD Sindh, 2015). For the demand-generation activities, Sindh looks to re-envision the role of dormant social institutions like social male mobilizers and also create new cadres of extension workers through male-led community-based organizations and male community elders who can promote health-seeking behaviors among young people and couples. The focus on men in Sindh’s CIP is to encourage them to support women’s use of family planning, with “the primary goal…to make the community aware of available services and motivate MWRA (married women of reproductive age) to obtain services.”

For supply-side activities, Sindh is focusing on provider training, referrals from extension agents, and the creation of youth-only spaces for education and community. The CIP focuses almost primarily on female methods of contraception. While the CIP projects increases in condom use from 8 percent of the method mix in 2011–12 to 10.5 percent in 2020, use of vasectomy as part of the method mix is projected to remain steady at 0.1 percent over the life of the CIP. The CIP projects only 1,012 adopters of vasectomy between 2014–2020, even though the province has invested in developing centers that provide no-scalpel vasectomy. Sindh will measure the effect of such programs with core FP2020 indicators which, to some degree, are male-specific and capture attitudinal as well as behavior change.

Sindh Province’s CIP does not acknowledge the deep and persistent gender norms that explain women’s limited access to family planning. While the CIP explicitly and consistently acknowledges the disadvantages that women live with in Sindhi society, it couches them as “social pressures,” “cultural norms,” or “lack of women’s empowerment” or as influenced by the “supportive outlook of the husband,” while not naming gender norms explicitly. Furthermore, they highlight factors that are symptomatic of patriarchal power structures, such as low mobility, lack of spousal communication, and poor knowledge. The CIP notes:

“In order to adapt to the social norms context in Sindh, there is a need to adopt innovative ways to facilitate use of FP services. As men usually go to work, and they are often reluctant to allow their wives to go to a facility and obtain reproductive health services on their own. It is therefore suggested that FP facilities may start an afternoon or evening shift—a time when male members would be at ease to bring their spouses to FP services—which would enhance access for both young couples as well as increase male engagement in FP” (p. 57).

The lack of acknowledgement of structural gender inequalities are reflected in the CIP: no policy and advocacy, nor financing and governance activities, are included that explicitly focus on men as users of reproductive health services or as partners or advocates for women’s use of reproductive health services.

### Senegal

Senegal’s commitment in 2012 mentioned increasing the involvement of men and young people along with leveraging networks leaders and champions to advocate for family planning. Senegal’s 2012–2105 plan, the *National Family Planning Action Plan 2012–2015,* includes a large-scale communication plan, mainly for men and youth, with tailored behavior change messages in favor of family planning and addressing misconceptions ( Republic of Senegal, 2012). Furthermore, the plan called for behavioral studies “to better define profiles and messages adapted to men and young people.” The plan also called for outreach activities “to sensitize [men] to the advantages of FP and widen the scope of FP to include the health of the couple.” Senegal’s 2016–2020 CIP, the *Cadre Strategique National de Planification Familiale 2016–2020* (National Strategic Framework for Family Planning 2016–2020) continues to characterize male engagement as a challenge ( Republique du Senegal, 2016). In particular, male knowledge is poor and attitudes towards family planning are often negative. Senegal’s 2016–2020 CIP describes men’s attitudes towards family planning as mixed, noting that a report from a communications campaign found that 50% of men ages 25–55 believe that people who use FP end up having health problems. As such, nearly all male engagement activities in Senegal’s FP plans are oriented toward demand generation and social and behavior change. The 2016–2020 plan called for strengthening communication directed towards men, building on earlier campaigns to help reluctant men and religious leaders to understand the benefits of family planning and improve their acceptance of family planning in homes and communities. Senegal employs a wide variety of channels to advance knowledge and perceptions of family planning among men: religious and community leaders, peer educators, grandfathers, science and home economics teachers, as well as mass media. Senegal also employs the Husbands’ Schools model ( IRH, 2020). Despite the range of demand generation and behavior change related activities, no service delivery interventions target men or couples. And while Senegal’s FP documents target male knowledge and attitudes towards family planning, there is no articulation of how gender norms will be addressed.

## Discussion and recommendations

In summary, while some of the country/sub-national plans that were analyzed did include aspects of male engagement, none addressed the issue comprehensively, as reflected in the definition of male engagement used in this paper. Across the plans, the strongest aspects were those that engaged men as supportive partners in order to promote women’s access to and use of family planning services, and improved men’s knowledge and attitudes related to contraception and reproduction. The weakest aspects were found in engaging men as users of family planning services and combatting the inequitable gender norms that prevent women and men from accessing the health services they need.

The international community has agreed that the right to health includes the right to control one’s health and body, including sexual and reproductive health ( UNCESCR, 2016). The full attainment of international family planning goals is not possible without men, who play significant roles in the lives of women and girls. Yet family planning programs often fail to take these relationships into account, or view men as uniformly ignorant or opposed to family planning use, reinforcing the notion that sexual and reproductive health and rights are a woman’s domain.

Before men and boys can be integrated into family planning programs, they must first be seen as relevant to efforts to increase contraceptive access for all, as deserving of the right to information and services, and as capable of wanting the same for their partners ( Greene *et al*., 2020). Yet the global frameworks and country plans highlighted in this paper fall short of fully addressing the role that inequitable gender dynamics and masculinities play in perpetuating poor sexual and reproductive health outcomes, a shortcoming which reinforces male power and exacerbates gender inequalities, ensuring that women continue to bear the responsibility for family planning, and leading to suboptimal health outcomes for both men and women. 

Our review of selected national and sub-national CIPs and action plans highlights that most activities working with men and boys are concentrated on demand generation. This finding is encouraging, as men should be engaged to play constructive roles in promoting gender equity and health in their families and communities. Yet, if implemented poorly, such programs may risk reinforcing existing gender hierarchies by elevating male voices at the expense of women’s and girls’. Everyone has a duty to recognize where women’s rights to autonomy and reproductive choices are not being respected: whether they are a doctor at a clinic doing couples counseling, a policymaker reforming protocols for the provision of services, or a husband discussing child spacing with his wife.

Equally important is to influence the institutions in places that enable effective male engagement as part of a larger strategy supportive of gender equality and women’s rights, such as governance structures, budget allotments, national/regional/local policies, and changing societal norms (such as those which silo FP as solely a woman’s issue and responsibility or the idea that bringing men and boys into FP will decrease women’s FP agency and decision-making). This also includes improving the way data are captured to provide gender-disaggregated information, and ensuring that the outcomes and outputs of family planning and reproductive health programs incentivize men’s engagement, where relevant.

Programs, policies and institutions that work towards goals that only include women’s access to family planning risk reinforcing the notion that men will be opponents to the goals of universal access to family planning. Therefore, just as it is important to cast a wide net in the range of ways men are encouraged to engage in family planning programs, it is also important not to treat men as a homogeneous group that is uniformly opposed to the use of family planning. Similarly, researcher-anthropologists should be encouraged to find intersectional approaches to engaging men with the understanding that people act in accordance with a constantly shifting set of influences and norms which often lean heavily on identity and culture, and that men may face significant marginalization not connected to their gender or sex that influence their abilities to be present partners and fathers.

Countrywide strategies related to FP2020 currently fall short of fully engaging men in family planning, including in meeting their own SRH needs. The recommendations below could bring about greater male engagement in country FP programs, and detail a way forward for the 2030 agenda. 

### Address harmful gender norms throughout the life-cycle

While the CIPs acknowledged cultural and gender barriers to family planning, some, such as Pakistan’s Sindh province, were indirect while others, such as Ethiopia’s were more direct, noting power and gender dynamics that inhibit women from making open decisions about FP use. Still, no CIP or action plan adequately addressed how it would confront harmful gender norms in its program descriptions. Rigid masculine norms around self-sufficiency impede men from positive health-seeking behaviors, with consequences for themselves, their partners, and gender equality more broadly ( Ragonese *et al.*, 2019). Interventions and initiatives throughout the entire life cycle should provide opportunities for reflecting on and challenging narrow gender roles and unequal power relations, and for practicing healthy, caring behaviors. This can be thought of as an *ethic*, which can be thoughtfully integrated across a country’s FP portfolio, particularly among programs for young people ( Blum *et al*., 2019), and must also not reinforce male hegemony in doing so. 

In many of the CIPs and action plans, “gender transformation” was not specifically included. For example, the majority of plans reviewed included commitments to building adolescent-friendly service sites, and still others had planned for training of providers in this approach. While adolescent-friendly services are one way to improve the state of adolescent sexual and reproductive health around the world, it is important that they be gender-responsive, treating boys and girls not as a homogenous population, but as having specific needs. Gender responsiveness means specifically naming boys (as well as girls) as a target group and addressing these concerns through a variety of means, such as ensuring that clinics guarantee anonymity, destigmatize care, and address structural-level factors like discriminatory policies. For example, due to rigid gender norm prescriptions, adolescent boys may require extra motivation to seek advice or visit a health provider and may be highly sensitive (i.e. unfamiliar, afraid) to the clinic environment once there. Importantly, any intervention to comprehensively address boys’ specific sexual and reproductive health concerns must be informed by gender-transformative approaches that deconstruct strict ideas of manhood ( Seth *et al*., 2020). The International Planned Parenthood Federation (IPPF) and UNFPA’s 2017 Global SRH Package for Men and Adolescent Boys ( Shand *et al*., 2017) points to some very specific steps clinics can take to ensure their youth-friendly services are also male-friendly. Other resources, including the Interagency Gender Working Group’s (IGWG’s) Do’s and Don’ts for Engaging Men and Boys ( Pulerwitz *et al*., 2019), and the High Impact Practice’s Engaging Men and Boys in Family Planning: A Strategic Planning Guide ( HIP, 2018) will also be of use to programmers. 

### Address male engagement explicitly through policy, standards and protocols

No CIP or action plan delineated explicit policy or advocacy goals to advance the engagement of men. Efforts targeting individual- and community-level change require supportive structural environments, as attitudes, behaviors, and access are shaped and influenced by institutions, policies, and the ways policies are implemented. Within a broader focus on men’s services and methods, such policies, norms and protocols could include training on vasectomy counseling, operation and post-operation for all family planning strategies, regulations, and policies, and a focus on male engagement in services proximal to family planning like antenatal care, post-partum care, and early childhood protocols and services which offer opportunities to include men at moments when the couple may be interested in discussing family planning. Similarly, male engagement in family planning is also complementary to policies to address the unequal burden of care work on women, and to involve men more deeply in parenting, through parental and paternity leave policies in the public and private sectors. This should complement an acceleration of research into safe, affordable and scalable male-controlled contraception.

### Implement couple-centered approaches

Too few demand generation and service delivery innovations in CIPs and action plans address partners together as contraceptive users. The Sindh province in Pakistan takes a couples-centered approach by encouraging health centers to open during evenings and weekends when men are off work. However, the Sindh province and other countries and states considering similar strategies should ensure that providers are trained to interact with and hear from both male and female partners during counseling, and to be aware of how men’s participation in FP counseling may undermine women’s voices, participation or autonomy. In patriarchal contexts, when men are present in such consultations, women may speak less, health care providers may focus more on the men as decision-makers, and show greater respect to men in FP consultations. In such cases, women’s decision-making and autonomy may be reduced by deference to or coercion by the male partner. Therefore, it is essential to ensure that providers address power dynamics within couple relationships to improve communication, while preserving the right of women to make their own SRH decisions at all times.

When conducted sensitively, couples counseling can be an effective means of improving a couple’s communication around family planning, challenging entrenched gender norms which prioritize male decision-making, and improving overall service uptake ( Doyle *et al*., 2018). The 2018 Guttmacher Lancet Commission on Sexual and Reproductive Health and Rights highlights this need for increased attention toward relational approaches to masculine norms and men as partners in sexual and reproductive health and rights ( Starrs *et al*., 2018).

### Develop men’s capacity as advocates and change agents for Sexual Reproductive Health and Rights

Too often, the CIPs and action plans were silent about the ways in which men were engaged as advocates. One possibility is that work with men is couched in other terms, such as working in conjunction with religious and community leaders, or youth groups. Explicitly engaging men in critical examinations of constructions of manhood is important to ensuring women’s empowerment and family planning access. Men, and particularly men in influential positions, are uniquely positioned to challenge these inequitable norms for the betterment of themselves and women’s health and equality ( Adams *et al.*, 2013; Greene *et al*., 2014; Singh *et al.*, 2014). Examples of male mobilizer programs, such as in Ethiopia, the Sindh Province in Pakistan, or Lagos State in Nigeria, point to the ways in which countries can train and organize groups of men for positive change. An evaluation of the Husbands’ Schools approach has found that it can contribute to increasing contraceptive use and transforming gender norms ( IRH, 2020).

### Include attitudinal and behavior change measures for men/boys in assessing program impact

We know that gender inequality undermines women’s family planning access. We also know that supportive, informed and engaged male partners can play an important role in increasing women’s access. Yet not one of the 18 core FP2020 indicators, collected across country programs, measures change in men’s attitudes or behaviors related to family planning use. As a result, too few CIPs and action plans track attitudinal or behavioral change related to gender norms and male engagement. This is incongruent with the numerous demand generation and social/behavior change programs spelled out in the CIPs and other documents. Expanding our measures of success to capture the gender-equality dividends of engaging men in SRHR can provide valuable data on pathways to increased healthcare utilization and improved health outcomes for women and men. Such measures could capture the benefits of improving couple communication and shared equitable decision-making, preventing gender-based violence, increasing men’s participation in prenatal visits, and expanding men’s involvement in maternal, newborn, and child health and caregiving.

For implementers or donors seeking inspiration on how to measure this, a list of 15 key indicators that family planning program implementers can use for monitoring and evaluating male engagement in FP programs was published by MEASURE Evaluation in 2018 ( Adamou *et al*., 2019).

### View men as users of contraceptive services, too

All programs included in this analysis focus contraceptive methods on women, with scant attention to reaching men as contraceptive users. Projections of contraceptive use over the life of the plans assume little or no increase in use of male methods or male cooperative methods, other than some attention to condoms. Evidence demonstrates men’s desire for information and services, and their positive response to existing programming ( Dorman & Bishai, 2012; Glasier, 2010; Heinemann *et al*., 2005; Kabagenyi *et al*., 2014; Lundgren *et al.*, 2012; Shattuck *et al*., 2016). Hardee *et al.* (2017) propose 10 considerations for the inclusion of men as FP users: provide information and services to men and boys where and when they need it; address gender norms that affect men’s use of contraceptive methods; meet men’s needs while respecting women’s autonomy; improve couple and community communication; link men’s contraceptive use with their desire to support their families; teach adolescent boys about pregnancy prevention and healthy sexual relationships; develop national policies and guidelines that include men as family planning users; scale up programs for men; fill the gaps through monitoring, evaluation, and implementation science; and create more contraceptive options for men. Research shows that if even 10 percent of men interested in using a new male-controlled method did so, the introduction of a male pill or temporary vas occlusion could have a substantial impact on pregnancy prevention, by 3.5 to 5.2 percent in the United States, 3.2 to 5 percent in South Africa, and as much as 30 to 38 percent in Nigeria ( Dorman *et al*., 2018).

## Conclusions

Evidence shows the importance of engaging men and boys in programs to reach the transformational goal of universal access to sexual reproductive health and rights, including family planning, for women and girls. Increasing the healthy, equitable, non-violent, and nurturing engagement of men and boys in family planning programs is central to improving health and wellbeing and reaching global goals for family planning and reproductive health. As we show in this paper, success rests also on understanding and accounting for the role of men and boys: as users of services, as partners for millions of women around the world, and as influencers in their communities. Strengthening male engagement in national plans would increase advances in family planning and contribute to achieving universal access by 2030. As FP2030 begins to take shape and countries design their next rounds of policies and strategic plans for family planning moving beyond 2020 to 2030, this review provides insights into how the inclusion of male engagement could be strengthened to improve women’s reproductive health and also to meet men’s needs for information and services. 

## Data availability

### Underlying data

All data underlying the results are available as part of the article and no additional source data are required.

### Extended data

Open Science Framework: A long way to go: engagement of men and boys in country family planning commitments and implementation plans

https://doi.org/10.17605/OSF.IO/MA6FB ( Hook, 2021)

This project contains the following underlying data:

- CIP Analysis Coding. (This dataset uses ‘counts’ for the number of times a keyword is utilized in a given document)

Data are available under the terms of the Creative Commons Zero "No rights reserved" data waiver (CC0 1.0 Public domain dedication).

## Footnotes

^i^ In 2020, the Department for International Development was replaced by the Foreign, Commonwealth & Development Office (FCDO).
