# Supplementary material for: A long way to go: engagement of men and boys in country family planning commitments and implementation plans
Source: Gates Open Res. 2021 Sep 27;5:85. Originally published 2021 May 21. [Version 2] doi: 10.12688/gatesopenres.13230.2 (PMC8187813; doi:10.12688/gatesopenres.13230.2)
Supplement: Peer review history

# Peer review: Reviewer response for version 2

**DOI:** 10.21956/gatesopenres.14642.r31214

**Published:** 2021-09-27

**Authors:** Ravi Verma

**Affiliations:** 1 Asia Regional Office, International Center for Research on Women (ICRW), New Delhi, Delhi, India

I am fine with the responses of the authors.

Is the work clearly and accurately presented and does it cite the current literature?

Yes

If applicable, is the statistical analysis and its interpretation appropriate?

Not applicable

Are all the source data underlying the results available to ensure full reproducibility?

Yes

Is the study design appropriate and is the work technically sound?

Yes

Are the conclusions drawn adequately supported by the results?

Yes

Are sufficient details of methods and analysis provided to allow replication by others?

Yes

Reviewer Expertise:

Gender and health especially in the areas of reproductive health, HIV and family planning; primary violence prevention; child marriage prevention; and engaging men and addressing masculine norms.

I confirm that I have read this submission and believe that I have an appropriate level of expertise to confirm that it is of an acceptable scientific standard.

# Peer review: Reviewer response for version 1

**DOI:** 10.21956/gatesopenres.14460.r30709

**Published:** 2021-06-21

**Authors:** Ravi Verma

**Affiliations:** 1 Asia Regional Office, International Center for Research on Women (ICRW), New Delhi, Delhi, India

By analyzing the costed implementation plan of seven African countries and one country in Asia the paper examines the extent to which these countries have responded to male engagement discourse in FP policies, programs, and resource commitments. One can reasonably assume that the CIPs and action plans would vary widely from one country to another in terms of the details and specificities It is also likely that the programs to engage men could be supported from the external financial support and could have been justified as an integral part of the strategy. In the absence of this information, which is perhaps beyond the scope of the paper, it is difficult to assess how far CIPs are the true reflections of the policy intentions and programs on the ground. Having said this, I think the paper is a timely reminder to the countries under analysis and to several other countries and their policymakers to pay attention to this important issue of male engagement in FP and prepare a comprehensive and explicit strategy.

Is the work clearly and accurately presented and does it cite the current literature?

Yes

If applicable, is the statistical analysis and its interpretation appropriate?

Not applicable

Are all the source data underlying the results available to ensure full reproducibility?

Yes

Is the study design appropriate and is the work technically sound?

Yes

Are the conclusions drawn adequately supported by the results?

Yes

Are sufficient details of methods and analysis provided to allow replication by others?

Yes

Reviewer Expertise:

Gender and health especially in the areas of reproductive health, HIV and family planning; primary violence prevention; child marriage prevention; and engaging men and addressing masculine norms.

I confirm that I have read this submission and believe that I have an appropriate level of expertise to confirm that it is of an acceptable scientific standard.

# Response

**Published:** 2021-09-13

**Authors:** Christopher Hook

By analyzing the costed implementation plan of seven African countries and one country in Asia the paper examines the extent to which these countries have responded to male engagement discourse in FP policies, programs, and resource commitments. One can reasonably assume that the CIPs and action plans would vary widely from one country to another in terms of the details and specificities It is also likely that the programs to engage men could be supported from the external financial support and could have been justified as an integral part of the strategy. In the absence of this information, which is perhaps beyond the scope of the paper, it is difficult to assess how far CIPs are the true reflections of the policy intentions and programs on the ground. Having said this, I think the paper is a timely reminder to the countries under analysis and to several other countries and their policymakers to pay attention to this important issue of male engagement in FP and prepare a comprehensive and explicit strategy.

**Response: **Thank you for this observation.  We agree that the stakeholders involved in developing the CIPs have been important in shaping the agendas for the plans.  As you note, it is beyond the scope of this manuscript to address this, but we encourage further analysis of this topic.  Perhaps a paper on the political economy of CIPs?

# Peer review: Reviewer response for version 1

**DOI:** 10.21956/gatesopenres.14460.r30712

**Published:** 2021-06-08

**Authors:** Bridgit M. Adamou

**Affiliations:** 1 Carolina Population Center, University of North Carolina at Chapel Hill, Chapel Hill, NC, USA

This paper looked at the extent to which male engagement in FP was incorporated into country or subnational FP goals. Based on the documents reviewed, there is limited inclusion of male engagement in FP. Doing so will strengthen existing FP programs and promote broader goals related to gender equality.

 Overall the paper was well-written and well-organized. My specific comments are as follows:

- Introduction, 2 ^nd^ paragraph: “Over the years, the vision for constructive male engagement in family planning…”, add the acronym “FP” here. Use the acronym hereafter.
- Methods, first bullet: The acronym “CIP” has already been introduced.
- The short section on Definition of male engagement needs clarification. Perhaps it would be more clear as follows:
  "Our definition of male engagement draws on a categorization that was developed by Greene *et al*. (2006) and adopted by the Interagency Gender Working Group. We define male engagement as:"
  "The intentional inclusion ...".
- Under Ethiopia, replace “Cyclebeads” with “standard days method”. (Cyclebeads are the tool used for the method.)
- Either replace “SRH” with “sexual and reproductive health”, as is mostly spelled out in the rest of the paper, or use “SRH” in the rest of the paper.
- Under Kenya, is “FH” supposed to be “RH”? If so, return to the Introduction, where reproductive health was first mentioned, introduce the acronym, and use RH thereafter.
- For some of the countries, indicators are mentioned (mostly, the lack of indicators for tracking male engagement), but not for all. I like how you included with Niger, “no indicators measure male engagement activities.” Because indicators are so meaningful to a program, if a CIP did not include indicators, I would state that as you did for Niger.
- Omit “In summary” from the beginning of the Discussion section. It gives the impression that the paper is concluding at this point.

Is the work clearly and accurately presented and does it cite the current literature?

Yes

If applicable, is the statistical analysis and its interpretation appropriate?

Not applicable

Are all the source data underlying the results available to ensure full reproducibility?

Yes

Is the study design appropriate and is the work technically sound?

Yes

Are the conclusions drawn adequately supported by the results?

Yes

Are sufficient details of methods and analysis provided to allow replication by others?

Yes

Reviewer Expertise:

Monitoring and evaluation of public health programs, family planning, male engagement, adolescent sexual and reproductive health, tuberculosis, indicator development, qualitative and quantitative data collection

I confirm that I have read this submission and believe that I have an appropriate level of expertise to confirm that it is of an acceptable scientific standard.

# Response

**Published:** 2021-09-13

**Authors:** Christopher Hook

This paper looked at the extent to which male engagement in FP was incorporated into country or subnational FP goals. Based on the documents reviewed, there is limited inclusion of male engagement in FP. Doing so will strengthen existing FP programs and promote broader goals related to gender equality.

 Overall the paper was well-written and well-organized. My specific comments are as follows:

- Introduction, 2 ^nd^ paragraph: “Over the years, the vision for constructive male engagement in family planning…”, add the acronym “FP” here. Use the acronym hereafter.

**Response:  **We have made these edits.

** **

- Methods, first bullet: The acronym “CIP” has already been introduced.

**Response:  **We have adjusted this. Because CIPs are the primary documents analyzed, we have left it spelled out here.

** **

- The short section on Definition of male engagement needs clarification. Perhaps it would be more clear as follows:
  "Our definition of male engagement draws on a categorization that was developed by Greene *et al*. (2006) and adopted by the Interagency Gender Working Group. We define male engagement as:"
  "The intentional inclusion ...".

**Response:  **We have revised this text based on this recommendation. 

** **

- Under Ethiopia, replace “Cyclebeads” with “standard days method”. (Cyclebeads are the tool used for the method.)

**Response: **Thank you for this reminder about SDM and Cyclebeads.  We have adjusted the text, using both terms since the method is more commonly known in countries by names such as Cyclebeads, moonbeads, etc.

** **

- Either replace “SRH” with “sexual and reproductive health”, as is mostly spelled out in the rest of the paper, or use “SRH” in the rest of the paper.

**Response: **We have made this edit to use SRH throughout, where appropriate.

** **

- Under Kenya, is “FH” supposed to be “RH”? If so, return to the Introduction, where reproductive health was first mentioned, introduce the acronym, and use RH thereafter.

**Response:  **Thank you for noting this. ****We have corrected it to be RH.

** **

- For some of the countries, indicators are mentioned (mostly, the lack of indicators for tracking male engagement), but not for all. I like how you included with Niger, “no indicators measure male engagement activities.” Because indicators are so meaningful to a program, if a CIP did not include indicators, I would state that as you did for Niger.

**Response:** We have added information about indicators in the sections for the other countries.

- Omit “In summary” from the beginning of the Discussion section. It gives the impression that the paper is concluding at this point.

**Response: **We have edited this accordingly.

# Peer review: Reviewer response for version 1

**DOI:** 10.21956/gatesopenres.14460.r30710

**Published:** 2021-06-07

**Authors:** Ilene S. Speizer

**Affiliations:** 1 Department of Maternal and Child Health, University of North Carolina Gillings School of Global Public Health, Chapel Hill, NC, USA

This paper provides a summary of how FP2020 country programs proposed addressing engagement of men and boys through a review of Costed Implementation Plans and Action Plans from seven countries, including some sub-national plans. The approach taken is comprehensive and provides excellent recommendations for future FP2030 country-level planning in support of male engagement. I only had a few minor comments that I elaborate below.

1. In the description of the analytic framework, it seems that the specifics of demand generation should include something targeted to men. As written, it focuses on general demand generation activities and does not specify those either targeted toward men as the audience or targeted with messages that promote male methods or couple/male engagement.
2. Also in the framework, the description of research, monitoring and evaluation misses the need for measures/measurement of male engagement. Further the “e.g.” on program quality does not necessarily cover quality – what are you trying to relay? Would a more appropriate male engagement quality indicator be the percent of providers trained on couple counseling? 
3. When you discuss youth friendly services, there is some assumption that this includes separate activities for female and male youth. It might be worth talking about this in the analytical framework section or at least describe what the assumptions are for “male engaged youth friendly services” that the review team was looking for.
4. I understand that the countries are presented in alphabetical order but I wondered if you thought about presenting them in an order of increasing male engagement (although as I read the paper a second time, it wasn’t completely obvious what that order would be – just a thought). 
5. In the Nigeria/National write up – it says “male engagement is nevertheless mentioned only once across all these activities.” I figured if it is only once, maybe you can elaborate and tell the reader how it is mentioned here. 
6. The statement at the bottom of page 7 (first column) seems inconsistent with the statement at the top of page 10 – page 7 statement: “Sindh will measure the effect of such programs with core FP2020 indicators which, to some degree, are male specific and capture attitudinal as well as behavior change.” – page 10 statement: “Yet not one of the 18 core FP2020 indicators, collected across country programs measures change in men’s attitudes or behaviors related to family planning use.”
7. In the discussion you state that most activities working with men and boys focus on demand generation activities. I wondered if this was similar to the overall CIPs (are they demand generation focused) or in contrast to the CIPs (are they supply focused)? Or perhaps the overall CIPs are balanced, so this is an indication of imbalance. 

Is the work clearly and accurately presented and does it cite the current literature?

Yes

If applicable, is the statistical analysis and its interpretation appropriate?

Not applicable

Are all the source data underlying the results available to ensure full reproducibility?

Yes

Is the study design appropriate and is the work technically sound?

Yes

Are the conclusions drawn adequately supported by the results?

Yes

Are sufficient details of methods and analysis provided to allow replication by others?

Yes

Reviewer Expertise:

I am a demographer/evaluation research who works on family planning/reproductive health so I am well prepared to review this paper.

I confirm that I have read this submission and believe that I have an appropriate level of expertise to confirm that it is of an acceptable scientific standard.

# Response

**Published:** 2021-09-13

**Authors:** Christopher Hook

This paper provides a summary of how FP2020 country programs proposed addressing engagement of men and boys through a review of Costed Implementation Plans and Action Plans from seven countries, including some sub-national plans. The approach taken is comprehensive and provides excellent recommendations for future FP2030 country-level planning in support of male engagement. I only had a few minor comments that I elaborate below.

 1. In the description of the analytic framework, it seems that the specifics of demand generation should include something targeted to men. As written, it focuses on general demand generation activities and does not specify those either targeted toward men as the audience or targeted with messages that promote male methods or couple/male engagement.

**Response:  **Thank you for this comment – we have adjusted the text accordingly.

 2. Also in the framework, the description of research, monitoring and evaluation misses the need for measures/measurement of male engagement. Further the “e.g.” on program quality does not necessarily cover quality – what are you trying to relay? Would a more appropriate male engagement quality indicator be the percent of providers trained on couple counseling? 

**Response:  **We have replaced the term “quality” with “output” – and have added something in the recommendations about measurement of male engagement.   

 3.  When you discuss youth friendly services, there is some assumption that this includes separate activities for female and male youth. It might be worth talking about this in the analytical framework section or at least describe what the assumptions are for “male engaged youth friendly services” that the review team was looking for. 

**Response:  **We have added youth friendly services in the analytic framework.  We found that while many CIPs mentioned YFS, they did not specify whether the services were for females and males.****

 4. I understand that the countries are presented in alphabetical order but I wondered if you thought about presenting them in an order of increasing male engagement (although as I read the paper a second time, it wasn’t completely obvious what that order would be – just a thought). 

**Response:  **Thank you for this suggestion.  We prefer to keep the list of countries in alphabetical order since we had not set criteria to measure strength of male engagement in CIPs in a way that would allow ranking the countries.****

****

 5. In the Nigeria/National write up – it says “male engagement is nevertheless mentioned only once across all these activities.” I figured if it is only once, maybe you can elaborate and tell the reader how it is mentioned here. 

**Response: **We have added the activity in the text. 

 6. The statement at the bottom of page 7 (first column) seems inconsistent with the statement at the top of page 10 – page 7 statement: “Sindh will measure the effect of such programs with core FP2020 indicators which, to some degree, are male specific and capture attitudinal as well as behavior change.” – page 10 statement: “Yet not one of the 18 core FP2020 indicators, collected across country programs measures change in men’s attitudes or behaviors related to family planning use.”

**Response:  **We have added indicators to the Sindh section and have removed the sentence about the FP2020 core indicators later in the paper.****

****

** 7. **In the discussion you state that most activities working with men and boys focus on demand generation activities. I wondered if this was similar to the overall CIPs (are they demand generation focused) or in contrast to the CIPs (are they supply focused)? Or perhaps the overall CIPs are balanced, so this is an indication of imbalance. 

**Response:**  Thank you for this good suggestion, which is outside the scope of the analysis we did.  This would make a good paper, particularly given the renewed attention to the need to address demand generation / social and behavior change as part of holistic family planning programming.
